# MM120 (lysergide) in a controlled clinical setting: treatment of generalized anxiety disorder without co-occurring psychotherapeutic intervention

**DOI:** 10.1192/j.eurpsy.2025.289

**Published:** 2025-08-26

**Authors:** S. M. Karas, R. Barrow, C. Conant, J. M. Freedman, P. L. Jacobsen, J. Jemison, D. R. Karlin, T. M. Solomon

**Affiliations:** 1 Mind Medicine, Inc., New York; 2 Department of Psychiatry, Tufts University School of Medicine, Boston, United States

## Abstract

**Introduction:**

Generalized anxiety disorder (GAD) is among the most common psychiatric disorders and has significant impairment on patients’ daily life. Effective and well-tolerated pharmacotherapies are needed. The FDA has noted that the inclusion of psychotherapy within trials complicates assessment of psychedelic drug efficacy, although to date many psychedelic trials have included co-occurring psychotherapy.^1^ Co-occurring psychotherapy confounds the evaluation of efficacy and may complicate commercial labeling. A phase 2b study of the dose response to single treatment MM120 (lysergide D-tartrate) suggests a safe, rapid, and durable dose-dependent response in participants with moderate-to-severe GAD^2^ and design elements are examined herein.

**Objectives:**

To examine the methodological use of Dosing Session Monitors (DSM) within MM120 treatment without co-occurring of psychotherapy.

**Methods:**

This phase 2b (NCT05407064) multicenter, randomized, double-blind, placebo-controlled study enrolled adults aged 18 to 74 years diagnosed with moderate to severe GAD.^2^ Two DSMs, meeting FDA recommended qualifications,^1^ were assigned to a participant throughout the study duration. Prior to dosing, participants engaged in baseline study education with their DSMs. During dosing, DSMs provided assistance and comfort as needed and monitored the participant for safety. Starting at 8 hours post-dose, a pre-specified checklist to end the session was administered, although all participants were required to stay onsite a minimum of 12 hours. The follow up period for the trial was 12 weeks, and DSMs only followed up with participants at Day 2, Week 1, and Week 2 post-dose. DSM follow up focused on participant safety and did not include focus on producing post-synaptic change.

**Results:**

In total, 198 participants were enrolled in the study. The phase 2b study demonstrated a statistically significant safe, dose-response relationship at the week 4, 8, and 12 primary and secondary endpoints.^1^ Utilizing the prespecified checklist, 45% of participants receiving MM120 100μg met criteria for end of session at 8 hours post dose, with 87.5% achieving criteria at 10 hours.

**Conclusions:**

The results demonstrate that a single treatment with MM120 produces a safe and durable clinical response without co-occurring psychotherapy. These findings indicate that MM120 treatment sessions may be safely shortened from a minimum of 12 hours to 8 hours for some participants, and psychotherapy does not appear to be essential for its safety and efficacy.

1. FDA—Center for Drug Evaluation and Research. Psychedelic drugs: considerations for clinical investigations (FDA-2023-D-1987).

2. Karlin, D et al. Rapid and Durable Response to a Single Dose of MM120 (Lysergide) in Generalized Anxiety Disorder: A Dose-Optimization Study. Poster P03-026. Presented at: APA Annual Meeting; May 4-8, 2024; New York City, NY. 2024.

**Disclosure of Interest:**

S. Karas Employee of: Mind Medicine, Inc., R. Barrow Employee of: Mind Medicine, Inc., C. Conant Employee of: Mind Medicine, Inc., J. Freedman Employee of: Mind Medicine, Inc., P. Jacobsen Employee of: Mind Medicine, Inc., J. Jemison Employee of: Mind Medicine, Inc., D. Karlin Employee of: Mind Medicine, Inc., T. Solomon Employee of: Mind Medicine, Inc.

